# Metformin enhances endogenous neural stem cells proliferation, neuronal differentiation, and inhibits ferroptosis through activating AMPK pathway after spinal cord injury

**DOI:** 10.1186/s12967-024-05436-9

**Published:** 2024-08-05

**Authors:** Cong Xing, Song Liu, Liyue Wang, Hongpeng Ma, Mi Zhou, Hao Zhong, Shibo Zhu, Qiang Wu, Guangzhi Ning

**Affiliations:** 1 Department of Orthopedics, Tianjin Medical University General Hospital, Tianjin, China; 2 International Science and Technology Cooperation Base of Spinal Cord lnjury, Tianjin, China; 3 Tianjin Key Laboratory of Spine and Spinal Cord Injury, Tianjin, China

**Keywords:** Spinal cord injury, Endogenous neural stem cells, Metformin, AMPK activation, Cell proliferation, Differentiation fate, Ferroptosis

## Abstract

**Background:**

Inadequate nerve regeneration and an inhibitory local microenvironment are major obstacles to the repair of spinal cord injury (SCI). The activation and differentiation fate regulation of endogenous neural stem cells (NSCs) represent one of the most promising repair approaches. Metformin has been extensively studied for its antioxidative, anti-inflammatory, anti-aging, and autophagy-regulating properties in central nervous system diseases. However, the effects of metformin on endogenous NSCs remains to be elucidated.

**Methods:**

The proliferation and differentiation abilities of NSCs were evaluated using CCK-8 assay, EdU/Ki67 staining and immunofluorescence staining. Changes in the expression of key proteins related to ferroptosis in NSCs were detected using Western Blot and immunofluorescence staining. The levels of reactive oxygen species, glutathione and tissue iron were measured using corresponding assay kits. Changes in mitochondrial morphology and membrane potential were observed using transmission electron microscopy and JC-1 fluorescence probe. Locomotor function recovery after SCI in rats was assessed through BBB score, LSS score, CatWalk gait analysis, and electrophysiological testing. The expression of the AMPK pathway was examined using Western Blot.

**Results:**

Metformin promoted the proliferation and neuronal differentiation of NSCs both in vitro and in vivo. Furthermore, a ferroptosis model of NSCs using erastin treatment was established in vitro, and metformin treatment could reverse the changes in the expression of key ferroptosis-related proteins, increase glutathione synthesis, reduce reactive oxygen species production and improve mitochondrial membrane potential and morphology. Moreover, metformin administration improved locomotor function recovery and histological outcomes following SCI in rats. Notably, all the above beneficial effects of metformin were completely abolished upon addition of compound C, a specific inhibitor of AMP-activated protein kinase (AMPK).

**Conclusion:**

Metformin, driven by canonical AMPK-dependent regulation, promotes proliferation and neuronal differentiation of endogenous NSCs while inhibiting ferroptosis, thereby facilitating recovery of locomotor function following SCI. Our study further elucidates the protective mechanism of metformin in SCI, providing new mechanistic insights for its candidacy as a therapeutic agent for SCI.

**Supplementary Information:**

The online version contains supplementary material available at 10.1186/s12967-024-05436-9.

## Graphical abstract

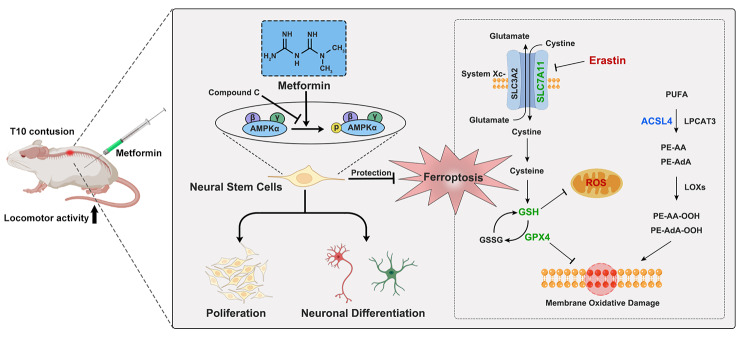

## Introduction

Spinal cord injury (SCI) inflicts devastating consequences on motor, sensory, and autonomic nervous functions, imposing significant burdens on patients, their families, and society [1]. Insufficient regeneration of neurons stands as a crucial barrier to repair SCI effectively [2]. Despite considerable progress has been made in pre-clinical and clinical research, it is evident that more targeted therapeutic interventions are needed [3]. Cell transplantation or activation of endogenous stem cells to replenish damaged neurons represents a highly promising therapeutic strategy for treating SCI [4–7]. However, the clinical application of cell transplantation is limited by ethical concerns, immune rejection reactions, and surgical complications. Conversely, the strategy of activating endogenous stem cells to supplement functional neurons appears to be safer and more feasible for clinical translation [4, 8, 9].

In adult mammalians, endogenous neural stem cells (NSCs) mainly reside in the subventricular zone, hippocampal subgranular zone, and the ependyma of the spinal cord [10–12]. NSCs are thought to have the potential to proliferate, self-renew and differentiate into neurons and glial cells [13]. However, the activation of NSCs after SCI is limited and they differentiate more into astrocytes than neurons [14–17]. Thus, the imperative lies in devising efficacious treatment modalities targeting the activation and differentiation fate regulation of endogenous NSCs.

Secondary injury caused by ischemia, hypoxia, lipid peroxidation, and inflammatory responses could lead to the formation of a locally inhibitory microenvironment, which is detrimental to the recruitment and survival of NSCs, further impeding endogenous neural regeneration [18, 19]. Recent studies suggested that ferroptosis, an iron-dependent programmed cell death form driven by lipid peroxidation, also plays an important role in secondary injury [20, 21]. The important hallmarks of ferroptosis include activity inhibition of glutathione peroxidase 4 (GPX4) and solute carrier family 7 member 11 (SLC7A11), upregulation expression of acyl-coenzyme A synthetase long-chain family member 4 (ACSL4), increased lipid peroxidation and disruption of mitochondrial structure [22]. Previous studies have reported that inhibiting iron overload in neurons and oligodendrocytes can regulate the imbalance of the microenvironment after SCI, thereby promoting the recovery of motor function [23–25]. However, whether neural stem cells undergo ferroptosis after SCI and their impact on neural regeneration remains to be elucidated.

Metformin is a widely used oral drug for type 2 diabetes mellitus, and AMP-activated protein kinase (AMPK) activation is its “classic” molecular pathway [26–28]. Besides its typical blood glucose-lowering effects, metformin is also extensively researched for its antioxidative, anti-inflammatory, anti-aging, and autophagy-regulating properties in central nervous system diseases like Alzheimer’s disease and Parkinson’s disease [29, 30]. Recent studies have shown that metformin promotes adult neurogenesis under normal physiological conditions and in various disease models [31–33]. Neuroprotective effects of metformin on neurons in vitro have also been reported [34]. Furthermore, metformin treatment markedly inhibits mitochondrial injuries and the expression of proteins and genes associated with ferroptosis in cardiomyocytes and neurons [35, 36]. However, conflicting findings suggest that metformin administration might exacerbate experimentally-induced acute kidney injury and slow tumor progression by promoting ferroptosis [37–39]. Therefore, further investigation is warranted to elucidate the precise mechanisms underlying the effects of metformin on NSCs.

To address these questions mentioned above, the primary aims of this study were to investigate the effects of metformin on NSCs activation and ferroptosis both in vitro and in vivo, and to determine whether these effects are mediated by canonical AMPK-dependent regulation.

## Materials and methods

### Animals

A total of 98 female Wistar rats aged 6–8 weeks and weighing 190–210 g were selected for in vivo experiments, and 8 pregnant Wistar rats at gestational days 12–14 were used for in vitro experiments. All rats were purchased from Charles River Laboratories. The rats were housed in SPF conditions at a temperature of 22 ± 2 ℃ and a humidity of 50-55%, with a 12-hour light-dark cycle. Food and water were provided ad libitum. The rats used for in vivo experiments were divided into five groups: Sham, Injury, CC, Met, and Met + CC (Supplementary Table 1). All animals were sacrificed by cervical dislocation under isoflurane anesthesia at the corresponding time points according to the experimental protocol. Experimental sections involving animals were approved by the Ethics Committee of the Institute of Radiation Medicine, Chinese Academy of Medical Sciences (IRM-DWLL-2,021,139) and were performed according to the guidelines of the National Institutes of Health.

### Spinal cord contusion model and drug intervention

The rats were randomly divided into 5 groups and anesthetized with isoflurane at an induction concentration of 2.5% and a maintenance concentration of 2.0%. The T10 spinal cord contusion model in rats was established using the MASCIS Impactor Model III (W. M. Keck Center, Rutgers University) and a modified Allen method. A laminectomy was performed at the T10 level, and a 10 g impactor was dropped from a height of 25.0 mm. After layer-by-layer suturing, the rats were placed on a heating pad for recovery. Cefotaxime (10 mg/kg) was intramuscularly injected daily for three days postoperatively to prevent infection. Bladder care was provided daily until the rats regained spontaneous urination. The sham group underwent laminectomy only without spinal cord injury. For the Injury group, normal saline was injected intraperitoneally daily postoperatively. For the Met group, metformin was injected intraperitoneally at a dose of 50 mg/kg daily postoperatively. For the CC group, compound C (CC) was injected intraperitoneally at a dose of 2 mg/kg daily postoperatively. For the Met + CC group, both metformin (50 mg/kg) and compound C (2 mg/kg) were injected intraperitoneally daily postoperatively.

### Chemicals

All chemicals used in the research were commercially available. Metformin, compound C (dorsomorphin), and erastin were purchased from Selleck Chemicals, Yuanye Biotechnology, and MedChemExpress, respectively. All chemicals were stored as stock solutions in phosphate-buffered saline (PBS) or dimethyl sulfoxide (DMSO).

### Isolation and culture of embryonic cortical NSCs

NSCs were harvested from the cerebral cortex of Wistar rat embryos at E13.5 as described previously with slight modification [40]. Briefly, rats were euthanized by isoflurane anesthesia followed by cervical dislocation. Then, the clipped cerebral cortexes were digested with 2 mg/ml papain (Solarbio) and 10 U/ml DNase I (Sigma) in DMEM medium (Gibco) for 10–20 min. The cell suspension was passed through a 40 μm cell filter and maintained in an uncoated T-75 flask with a growth medium. The NSCs-specific growth medium was formulated with DMEM medium (Gibco) with 2% B27 supplements without Vitamin A (Gibco), 1% GlutaMAX (Gibco), 1% penicillin/streptomycin (Gibco), 20 ng/ml bFGF (R&D systems), and 20 ng/ml EGF (R&D systems). The medium was half replaced every other day and cells were passaged with Accutase (Sigma) every 7 days and used for subsequent experiments after 2–4 passages.

Before each test was performed, NSCs were digested and seeded on plates precoated with 0.01% poly-L-lysine and 2 ug/ml laminin (Sigma) so that they would grow as single cells rather than neurospheres state. In addition, prior to the cell differentiation assay, the medium was replaced with the differentiation medium consisting of DMEM/F-12 medium, 2% B27 supplement with vitamin A (Gibco), 1% GlutaMAX, and 1% penicillin/streptomycin.

### Immunofluorescence staining

NSCs and 10 μm spinal cord slices were fixed with 4% paraformaldehyde for 15 min, followed by TBST washing three times. Subsequently, samples were blocked and permeabilized with 5% bovine serum albumin (Solarbio) and 0.25% Triton X-100 (Beyotime) for 1.5 h and then incubated overnight at 4℃ with primary antibodies: Nestin (R&D systems, MAB2736), SOX2 (Abcam, ab93689), Tuj-1 (Abcam, ab18207), GFAP (Cell Signaling Technology, 3670), Olig2 (R&D systems, AF2418) and GPX4 (Abcam, ab125066). After TBST washing three times, samples were incubated for 1 h with appropriate secondary antibodies (Supplementary Table 2). The nuclei were stained with DAPI. Immunofluorescence images were captured using a Zeiss LSM 800 confocal microscope or a Leica DMi8 fluorescence microscope. All fluorescence images were processed and calculated using Image J (version 1.8.0, NIH).

### CCK-8 assay

Cell viability was determined by CCK-8 assay (Dojindo). Briefly, NSCs were incubated in growth medium with 10% CCK-8 working buffer for 2–4 h. Then absorbance at 450 nm was measured using a Synergy HTX multi-mode reader (Biotek).

### EdU staining

Cell proliferation was determined by EdU assay kit (Beyotime). Briefly, NSCs were treated with 10 µM EdU working buffer for 24 h. Following fixing with 4% paraformaldehyde for 15 min and permeabilizing with PBS containing 0.3% Triton X-100 for 10 min, cells were incubated with 200 µL click reaction solution for 30 min. Images were obtained and analyzed using a Leica DMi8 fluorescence microscope and ImageJ software.

### Western blot

NSCs and spinal cord tissues were homogenized in RIPA lysis buffer (Beyotime). Proteins were separated by 10% SDS-PAGE and transferred to a PVDF membrane (Millipore). Following blocking with 5% skimmed milk (BD) for 1 h, membranes were incubated overnight at 4 ℃ with primary antibodies: p-AMPK (Cell Signaling Technology, 2535), AMPK (Cell Signaling Technology, 2793), ACSL4 (Abcam, ab155282), SLC7A11 (Abcam, ab175186), GPX4 (Abcam, ab125066) and GAPDH (Proteintech, 10494-1-AP). Then membranes were incubated for 1 h with secondary antibodies (Supplementary Table 2). Protein bands were quantified using ImageJ software. GAPDH was used as the internal standard. Specially, phosphorylation levels of AMPK were quantified by calculating the band intensity ratio of p-AMPK and AMPK.

### ROS detection

The intracellular ROS levels were determined by the DCFH-DA assay kit (MedChemExpress). Briefly, NSCs were incubated at 37℃ for 15 min in PBS with 10 µM DCFH-DA solution, followed by PBS washing three times. Images were obtained using a Leica DMi8 fluorescence microscope, and the fluorescence intensity was analyzed using ImageJ software.

### GSH detection

The intracellular GSH levels were determined by a Reduced Glutathione assay kit (Solarbio). Briefly, each sample was counted and homogenized in 1 mL extraction solution by freezing and thawing three times. After centrifugation at 8,000 g for 10 min, the supernatants were used for assays, and absorbance at 412 nm was measured using a microplate reader.

### Mitochondrial membrane potential (MMP) assay

The MMP was determined by the JC-1 assay kit (Solarbio) following the manufacturer’s instructions. Briefly, NSCs were treated for 20 min with JC-1 dye (1:200), followed by wash buffer washing three times. Images were obtained and analyzed using a Leica DMi8 fluorescence microscope and ImageJ software, respectively. The MMP was represented by the ratio between red (aggregates) and green (monomers) fluorescence intensity.

### Transmission electron microscopy

Differently treated NSCs were collected and fixed in PBS with 2.5% glutaraldehyde at 4 °C. Following gradient dehydration through different concentrations of alcohol, samples were penetrated with resin, embedded, and then polymerized using a cryogenic UV polymerizer (Electron Microscopy China). 80 nm ultrathin Sect. (80 nm) were obtained using an ultramicrotome (EM UC7, Leica) and transferred to a nickel mesh. Images available for observation of mitochondrial structure were obtained by transmission electron microscopy (HT 7700, Hitachi).

### Basso, Beattie, and Bresnahan (BBB) locomotor rating scale

The recovery of hind limb motor function of rats was assessed using the BBB scale, ranging from 0 to 21 points. The main evaluation indicators included joint mobility (hip, knee, and ankle joints), weight-bearing movement, coordination of forelimb and hindlimb movements, toe clearance, paw position, tail position, and trunk stability. Assessments were conducted on days 3, 7, 14, 21, 28, 42, and 56 post-injury, with all rats evaluated in a spacious and quiet environment, allowing each rat to freely move for 4 min. The assessments were performed by two experienced personnel who were blinded to the grouping. In cases of disagreement between the two raters, a discussion was held to reach a consensus, and the final score was recorded accordingly.

### Louisville swim scale (LSS)

At 56 days post-injury, all rats underwent swimming tests with scores ranging from 0 to 17 points. The assessment primarily focused on hind limb movement and alternation, forelimb dependency, trunk instability, and body angle. A glass tank measuring 60 cm × 33 cm × 38 cm was used as the swimming pool, with water maintained at a temperature of 24–25 ℃ and a depth of approximately 30 cm. Rats were placed in the pool for testing, with each rat’s test duration lasting 4 min. Two experienced personnel, unaware of the groupings, conducted the scoring and recorded the tests simultaneously. In cases of discrepancy between the two raters, the average score was taken. After completion of the experiment, rats were dried and placed on a heating pad to maintain body temperature.

### CatWalk gait analysis

Gait analysis in rats at 56 days post-injury was performed using the CatWalk XT automatic gait analysis system (version 10.6, Noldus) [41]. The parameters were set as follows: camera gain: 17.5 dB, green walkway light: 15.4 V, red ceiling light: 17.1 V, green intensity threshold: 0.12, and time threshold set from 0.5 s to 15 s. In a dimly lit environment, rats were individually placed on one side of the glass walkway, with food placed on the other side to guide them through. A pass without pause by the rat was recorded as one run. Each rat passed the walkway three times, and after successfully obtaining three sets of data, the parameters were merged for further analysis.

### Electrophysiological testing

Nerve conduction function of rats at 56 days post-injury was assessed using electrophysiological equipment (YRKJ-G2008, Zhuhai Yiriki Co., Ltd.). After anesthesia with isoflurane, stimulating electrodes were inserted subcutaneously between the ears of the rats, reference electrodes were inserted subcutaneously on their backs, and recording electrodes were inserted into the bilateral gastrocnemius muscles. Motor evoked potentials (MEP) were elicited by 10 mA current stimulus. Each rat underwent three repeated tests, and waveforms were recorded and saved.

### Tissue iron detection

The spinal cord iron content was determined by the tissue iron content assay kit (Solarbio) following the manufacturer’s instructions. Briefly, 50 mg of spinal cord tissue from the injury site was harvested at 7 days post-injury, and each sample was homogenized in 1 mL extraction solution. After centrifugation at 4,000 g for 10 min, the supernatants were used for assays, and absorbance at 520 nm was measured using a microplate reader.

### Hematoxylin-eosin (HE) and nissl staining

At 56 days post-injury, spinal cord and bladder tissues were subjected to HE or Nissl staining using the Hematoxylin-Eosin Stain Kit (Solarbio) or Nissl Stain Kit (Solarbio). Rats subjected to cardiac perfusion after isoflurane overdose anesthesia to collect spinal cord and bladder tissues. After fixation, dehydration, and embedding, the samples were sliced into 10 μm thick sections. Following the manufacturer’s instructions, the sections were stained with hematoxylin and eosin or cresyl violet, and photographed under a microscope. Pictures were processed and analyzed using Image J (version 1.8.0, NIH).

### Statistical analysis

Statistical analysis was performed with GraphPad Prism (version 8.0.1). Two groups were compared with an unpaired two-tailed *t* test. The comparison of BBB scores was conducted using two-way ANOVA with Dunnett’s post-hoc test. Other multiple comparisons were evaluated by one-way ANOVA followed by Dunnett or Tukey’s post-hoc test. Data were presented as the mean ± SEM, and *p* values < 0.05 were considered statistically significant.

## Results

### Metformin enhances the proliferation and neural differentiation of NSCs

Primary NSCs were isolated from the cerebral cortex of E12-14 Wistar rat embryos and subsequently proliferated and aggregated to form neurospheres in the growth medium (Supplementary Fig. 1A, B). These cells were co-stained with the NSCs-specific markers SOX2 and Nestin in both the neurospheres and single-cell states (Supplementary Fig. 1C). The differentiation potential of NSCs was evaluated on day 7 after changing to the differentiation medium and culturing in the single-cell state. Different proportions of Tuj-1, GFAP, and Olig2 positive cells can be observed by immunofluorescence staining (Supplementary Fig. 1D). These results suggested that rat embryonic cortical-derived NSCs could proliferate and differentiate into neurons, astrocytes, and oligodendrocytes in vitro.

**Fig. 1:**
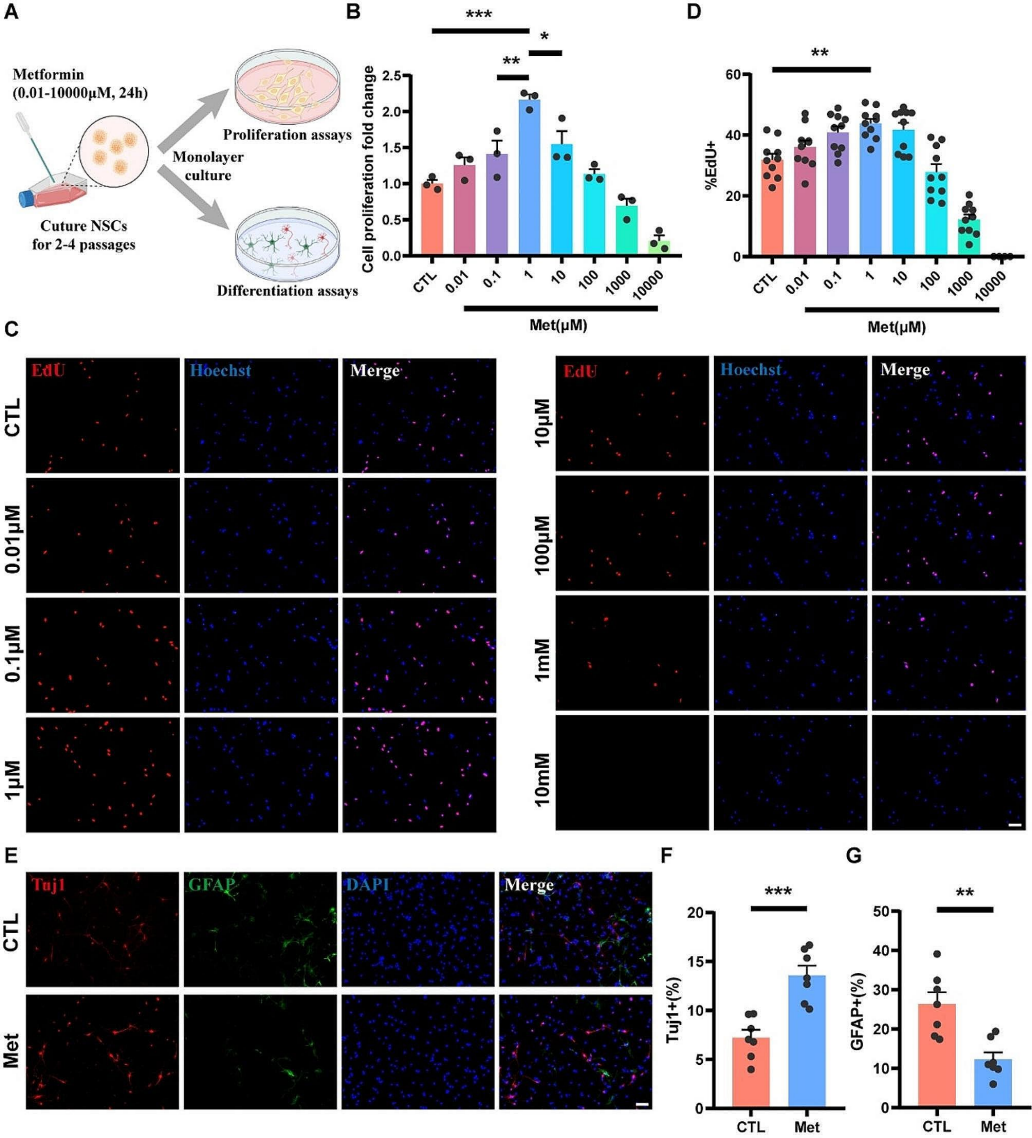
Metformin enhances the proliferation and neural differentiation of NSCs. (**A**) Schematic diagram of the analysis of primary NSCs treated with different concentrations of metformin for 24 h. (**B**) Cell viability assay of NSCs treated with different concentrations of metformin (*N* = 3). Cells were cultured in NSCs-specific growth medium for 24 h before assessment. The cell viability was calculated as fold changes of the control group. (**C, D**) Representative immunofluorescence images of EdU (red) positive cells and quantitative analysis of the proportions of EdU positive cells in NSCs (*N* = 10). Cells were cultured in NSCs-specific growth medium for 24 h before assessment. Cell nuclei were stained with Hoechst (blue). Scale bar = 50 μm. (**E-G**) Representative immunofluorescence images of NSCs double-labeled with Tuj-1 (red) and GFAP (green), and quantitative analysis of the respective proportions of Tuj-1 positive cells and GFAP positive cells in NSCs (*N* = 7). Cells were cultured in NSCs differentiation medium for 7 d before assessment. Cell nuclei were stained with DAPI (blue). Scale bar = 50 μm. A and C were analyzed using one-way ANOVA and Tukey’s post-hoc test, E and F were analyzed using unpaired t-tests. Data are shown as mean ± SEM. (* *p* < 0.05, ** *p* < 0.01, *** *p* < 0.001)

To investigate the potential effects of metformin on NSCs, we observed the changes in proliferation and differentiation capacity (Fig. 1A). CCK-8 assay showed that cell viability of NSCs increased after 0.01 ∼ 100 µM metformin treatment for 24 h, and the highest viability was observed with 1 µM metformin treatment. (Fig. 1B). Similar results were obtained in EdU staining, the proportion of EdU-positive cells was significantly increased after 1 µM metformin treatment for 24 h compared to the control group (43.80 ± 1.55%, 31.92 ± 1.95%, respectively; *p* < 0.01) (Fig. 1C, D). It is worth noting that excessive concentrations (1000 µM and 10,000 µM) of metformin were found to inhibit the proliferation of NSCs in both assays. Next, we sought to evaluate the effect of metformin on the differentiation fate of NSCs by immunofluorescence staining. NSCs were changed to culture in the differentiation medium and treated with 1 µM metformin for 24 h, Tuj-1 and GFAP were used as specific markers of neurons and astrocytes to evaluate changes in the proportion of differentiated cells. The results showed that compared with the control group, the proportion of Tuj-1 positive cells increased significantly (13.58 ± 0.99%, 7.22 ± 0.80%, respectively; *p* < 0.001) with metformin treatment, and the proportion of GFAP positive cells was significantly decreased (12.27 ± 1.80%, 26.37 ± 3.03%, respectively; *p* < 0.01) (Fig. 1E-G). Collectively, these findings suggested that treatment of 1 µM metformin could enhance the proliferation and neural differentiation of rat embryonic cortical NSCs in vitro.

### The beneficial effects of metformin on NSCs are driven by the AMPK-dependent regulation

**Fig. 2:**
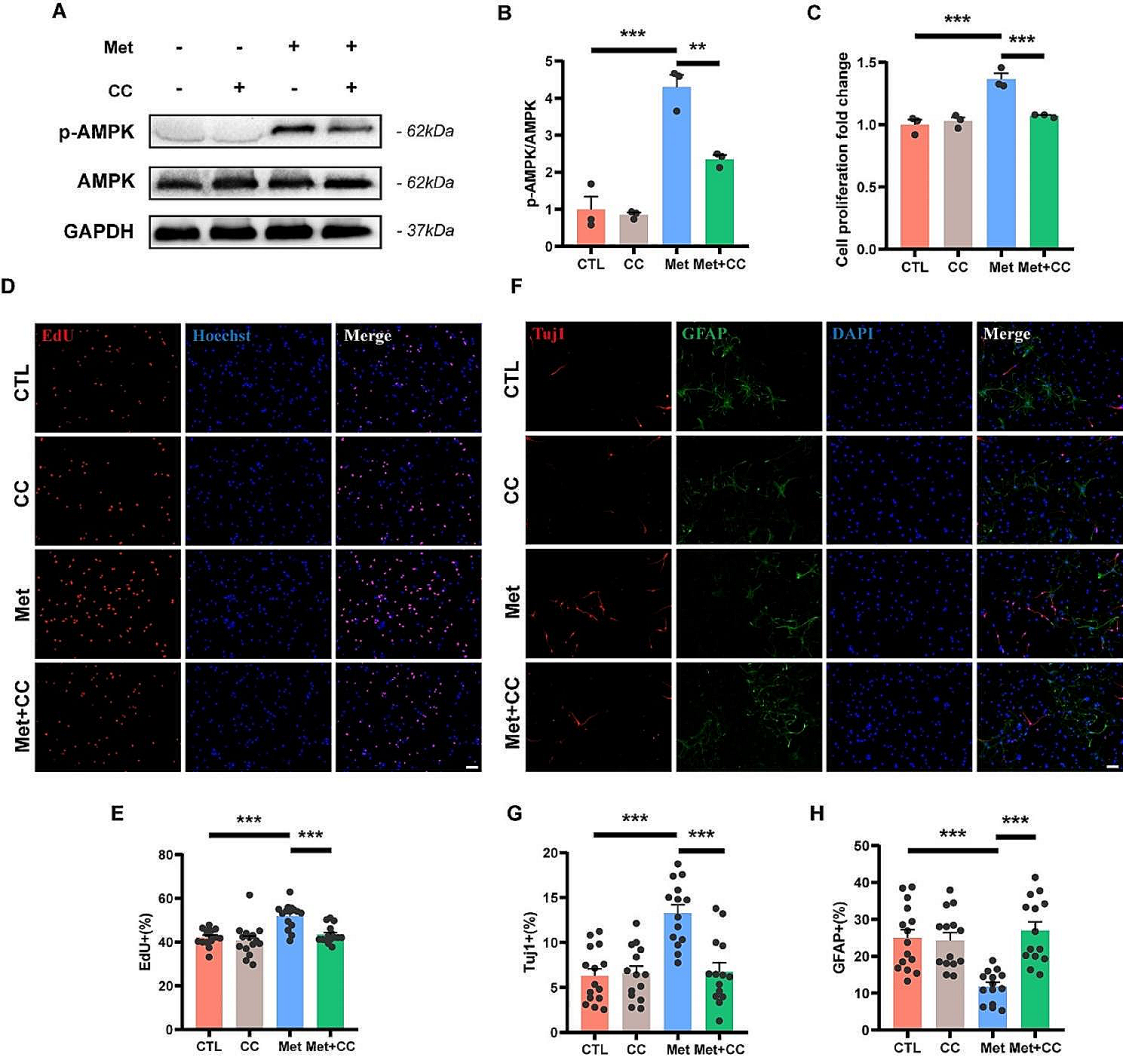
Metformin enhances NSCs proliferation and neural differentiation via AMPK-dependent regulation. (**A**, **B**) Representative western blot images of p-AMPK and AMPK and quantitative analysis of p-AMPK / AMPK expression level of NSCs with different treatments (*N* = 3). The relative expression level of target proteins was normalized by GAPDH and then calculated as fold changes of the control group. (**C**) Cell viability assay of NSCs with different treatments (*N* = 3). Cells were cultured in NSCs-specific growth medium for 24 h before assessment. The cell viability was calculated as fold changes of the control group. (**D, E**) Representative immunofluorescence images of EdU (red) positive cells and quantitative analysis of the proportions of EdU positive cells in NSCs (*N* = 15). Cells were cultured in NSCs-specific growth medium for 24 h before assessment. Cell nuclei were stained with Hoechst (blue). Scale bar = 50 μm. (**F-H**) Representative immunofluorescence images of NSCs double-labeled with Tuj-1 (red) and GFAP (green), and quantitative analysis of the respective proportions of Tuj-1 positive cells and GFAP positive cells in NSCs (*N* = 14). Cells were cultured in NSCs differentiation medium for 7 d before assessment. Cell nuclei were stained with DAPI (blue). Scale bar = 50 μm. All analyses were performed using one-way ANOVA and Dunnett’s post-hoc test. Data are shown as mean ± SEM. (* *p* < 0.05, ** *p* < 0.01, *** *p* < 0.001)

Next, we examined the changes in AMPK protein expression. As can be seen from the results of the western blot, the phosphorylation level of AMPK in metformin-treated NSCs was increased compared to the control group (Supplementary Fig. 2A, B). In order to determine whether the effects of metformin on NSCs depend on the activation of the AMPK pathway, compound C was used to specifically inhibit AMPK activation. The maximum safe concentration of compound C on NSCs was 1 µM by cell viability assay (Supplementary Fig. 2C), and the results of western blot confirmed that 1 µM compound C significantly inhibited metformin-mediated AMPK phosphorylation (Fig. 2A, B). The CCK-8 assay showed significantly lower cell viability in the Met + CC group compared to the Met group (Fig. 2C). Similar results were observed in EdU staining, with a significantly lower percentage of EdU positive cells in the Met + CC group than in the Met group (43.45 ± 1.04%, 51.95 ± 1.47%, respectively; *p* < 0.001) (Fig. 2D, E). In addition, immunofluorescence staining showed significantly lower Tuj-1 positive cells in the Met + CC group than in the Met group (6.76 ± 0.98%, 13.28 ± 0.94%, respectively; *p* < 0.001) (Fig. 2F, G). In contrast, GFAP-positive cells were significantly higher in the Met + CC group than in the Met group (26.99 ± 2.35%, 11.78 ± 1.16%, respectively; *p* < 0.001) (Fig. 2F, H). Furthermore, the addition of compound C alone did not significantly alter the proliferation capacity and differentiation fate of NSCs. The aforementioned experimental results suggest that inhibition of AMPK activation counteracts the effects of metformin on the proliferation and neural differentiation of NSCs. Thus, these beneficial effects are driven by AMPK-dependent regulation.

### Erastin induces NSCs ferroptosis

**Fig. 3:**
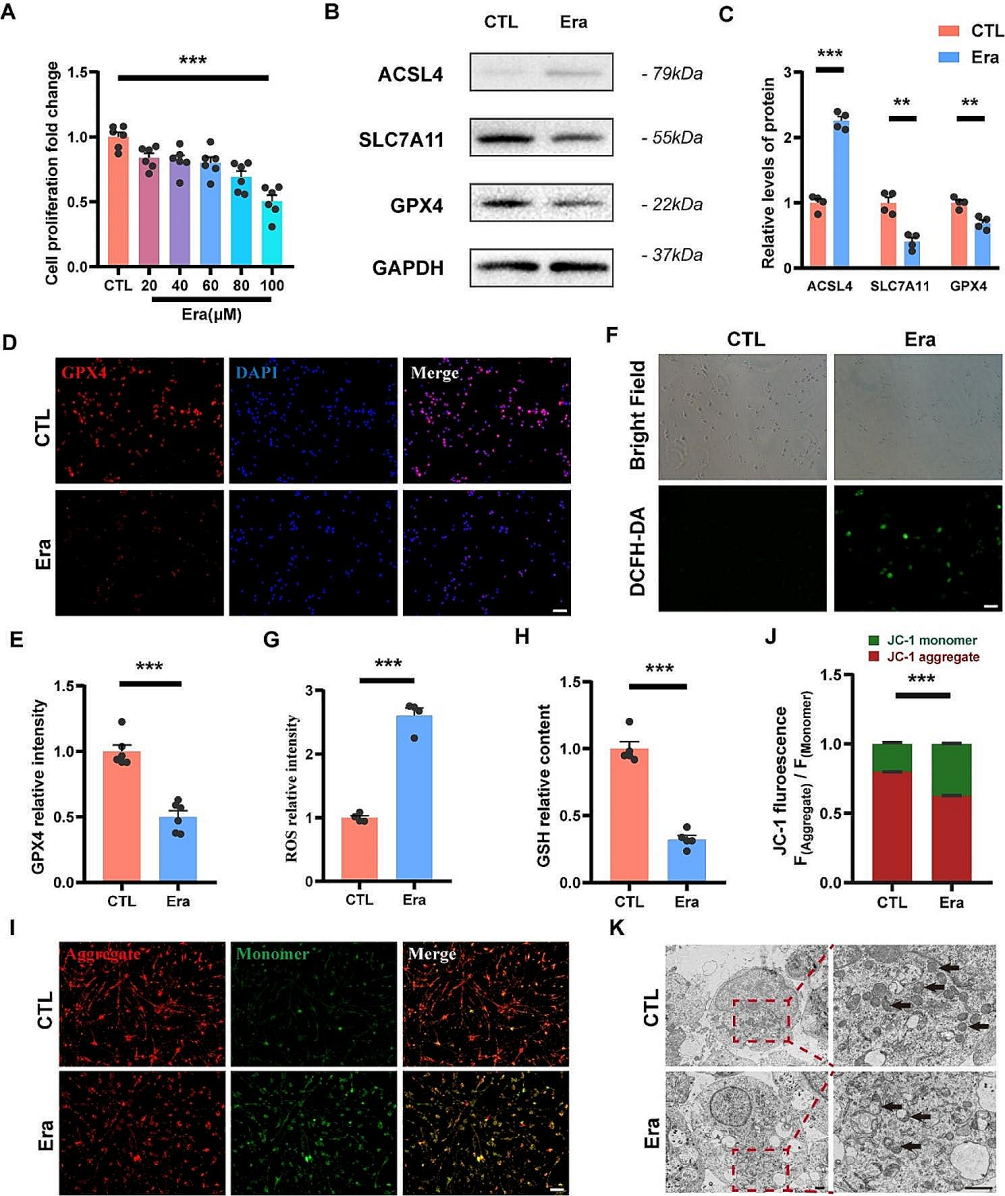
Erastin induces NSCs ferroptosis. (**A**) Cell viability assay of NSCs treated with different concentrations of erastin (*N* = 5). The cell viability was calculated as fold changes of the control group. (**B, C**) Representative western blot images and quantitative analysis of expression level of ACSL4, SLC7A11 and GPX4 in erastin-treated and untreated NSCs (*N* = 4). The relative expression level of target proteins was normalized by GAPDH and then calculated as fold changes of the control group. (**D, E**) Representative immunofluorescence images of NSCs labeled with GPX4 (red), and quantitative analysis of the relative fluorescence intensity of GPX4 (*N* = 6). Cell nuclei were stained with DAPI (blue). The relative fluorescence intensity was calculated as fold changes of the control group. Scale bar = 50 μm. (**F, G**) Representative images of DCFH-DA and quantitative analysis of the relative fluorescence intensity of ROS in NSCs (*N* = 4). The relative fluorescence intensity was calculated as fold changes of the control group. Scale bar = 50 μm. (**H**) Quantitative analysis of GSH expression level of NSCs (*N* = 5). The relative GSH content was calculated as fold changes of the control group. (**I, J**) Representative immunofluorescence images of JC-1 and quantitative analysis of the relative fluorescence intensity ratios of aggregate (red) / monomer (green) (*N* = 6). Scale bar = 50 μm. (**K**) Representative transmission electron microscopy images of NSCs and mitochondria were indicated by black arrows. Scale bar = 1 μm. A was analyzed using one-way ANOVA with Dunnett’s post-hoc test, and all other analyses were conducted using unpaired t-tests. Data are shown as mean ± SEM. (* *p* < 0.05, ** *p* < 0.01, *** *p* < 0.001)

To investigate whether metformin could exert a neuroprotective effect against ferroptosis in NSCs, we attempted to develop a ferroptosis model of NSCs with erastin in vitro. The CCK-8 assay showed a progressive decrease in cell viability of NSCs with exposure to different concentrations of erastin (20, 40, 60, 80, 100 µM) for 24 h compared to the control, and a nearly 50% decrease in cell viability with 100 µM erastin treatment (Fig. 3A). In subsequent experiments, we chose a concentration of 100 µM erastin to treat the NSCs to induce ferroptosis. A significant decrease in the expression levels of GPX4 and SLC7A11 and a significant increase in the expression level of ACSL4 were observed in NSCs treated with erastin (Fig. 3B, C). A similar result was observed in the immunofluorescence staining results, the relative fluorescence intensity of GPX4 in NSCs treated with erastin was also significantly decreased (Fig. 3D, E). The changes in the expression of these key proteins are consistent with the characteristics of cell ferroptosis. The results of DCFH-DA fluorescence probe assay and GSH content assay indicated that erastin treatment caused an increase in ROS production and a decrease in GSH content in NSCs, respectively (Fig. 3F-H). Moreover, the ratio of aggregates/monomers in JC-1 staining was decreased in the erastin group compared with the control group, indicating that the mitochondrial membrane potential of erastin-treated NSCs has downregulated (Fig. 3I, J). Morphological abnormalities of mitochondria in erastin- treated NSCs were also observed in transmission electron microscopy images (Fig. 3K). The above results indicate that treatment with 100µM erastin for 24 h is effective in inducing ferroptosis of NSCs in vitro.

### Metformin inhibits ferroptosis in NSCs via AMPK-dependent regulation

**Fig. 4:**
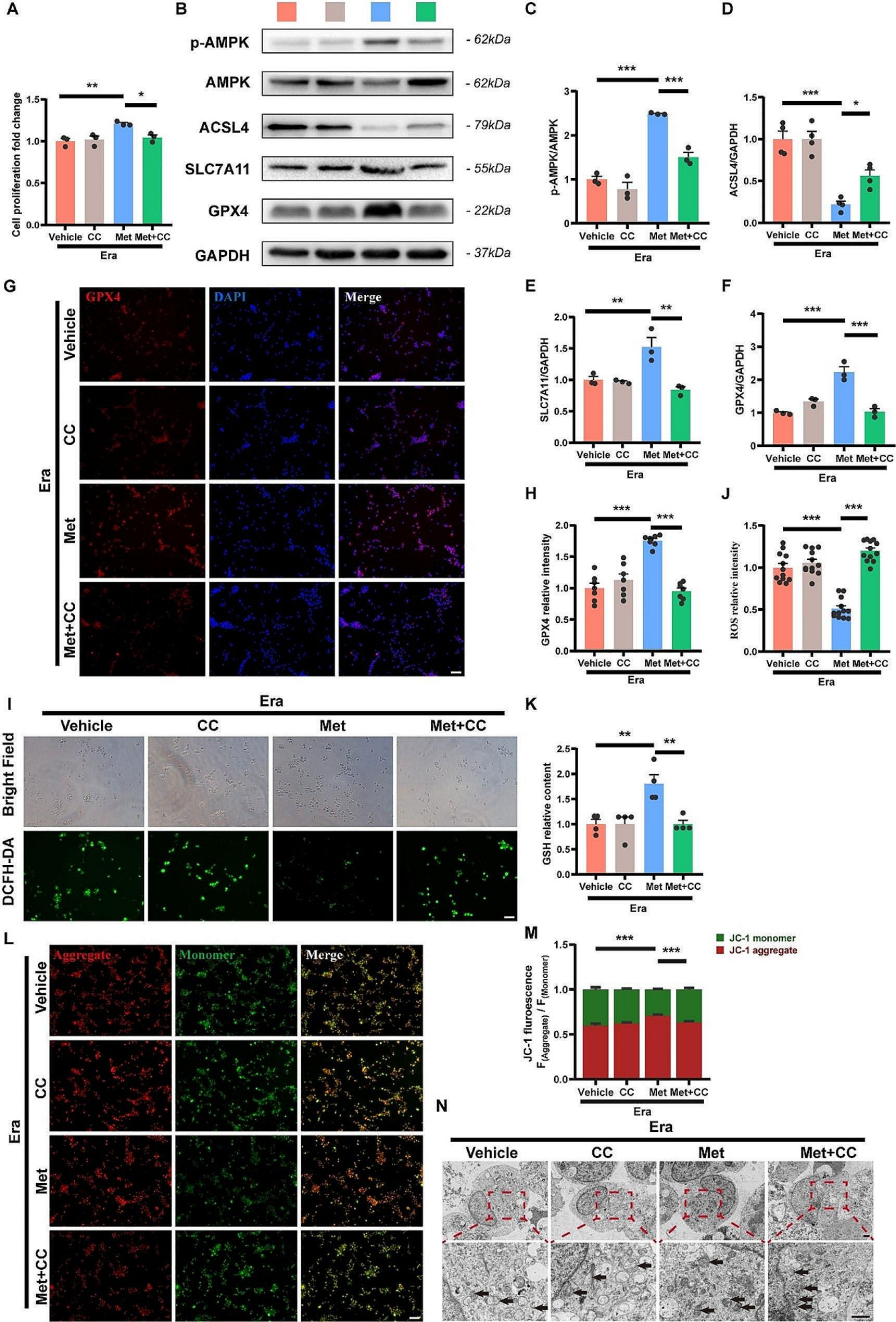
Metformin inhibits erastin-induced ferroptosis in NSCs via AMPK-dependent regulation. (**A**) Cell viability assay of NSCs with different treatments (*N* = 3). The cell viability was calculated as fold changes of the erastin group. (**B-F**) Representative western blot images and quantitative analysis of expression level of ACSL4, SLC7A11 and GPX4 in NSCs (*N* = 3). The relative expression level of target proteins was normalized by GAPDH and then calculated as fold changes of the erastin group. (**G, H**) Representative immunofluorescence images of NSCs labeled with GPX4 (red), and quantitative analysis of the relative fluorescence intensity of GPX4 (*N* = 7). Cell nuclei were stained with DAPI (blue). The relative fluorescence intensity was calculated as fold changes of the erastin group. Scale bar = 50 μm. (**I, J**) Representative images of DCFH-DA and quantitative analysis of the relative fluorescence intensity of ROS in NSCs (*N* = 12). The relative fluorescence intensity was calculated as fold changes of the erastin group. Scale bar = 50 μm. (**K**) Quantitative analysis of GSH expression level of NSCs (*N* = 4). The relative GSH content was calculated as fold changes of the erastin group. (**L, M**) Representative immunofluorescence images of JC-1 and quantitative analysis of the relative fluorescence intensity ratios of aggregate (red) / monomer (green) (*N* = 10). Scale bar = 50 μm. (**N**) Representative transmission electron microscopy images of NSCs. Mitochondria were indicated by black arrows. Scale bar = 1 μm. All analyses were conducted using one-way ANOVA with Dunnett’s post-hoc test. Data are shown as mean ± SEM. (* *p* < 0.05, ** *p* < 0.01, *** *p* < 0.001)

Next, we observed the effects of metformin on erastin-induced ferroptosis in NSCs and explored whether the effects were also regulated by the activation of AMPK. It was found that the addition of 0.1 µM, 1 µM and 10 µM metformin increased the cell viability of erastin-treated NSCs, while the optimal concentration of 1 µM was consistent with the enhancement of NSCs proliferation and neuronal differentiation (Supplementary Fig. 3A). And the improved survival of erastin-treated NSCs by metformin was counteracted by the addition of compound C (Fig. 4A). Metformin treatment reversed erastin-induced changes in the expression levels of GPX4, SLC7A11, and ACSL4 in NSCs (Fig. 4B, D-F), and the relative fluorescence intensity of GPX4 also increased correspondingly (Fig. 4G, H). Similarly, Compound C inhibited metformin-induced phosphorylation of AMPK and inhibited changes in the expression level of key proteins of ferroptosis (Fig. 4B-H). Notably, there was no significant change in the expression of these key proteins of ferroptosis when NSCs were treated with metformin intervention alone (Supplementary Fig. 3B-E). The results of the DCFH-DA fluorescence probe assay and GSH content assay reflected the same trend, with the addition of metformin reversing the erastin-induced increase in ROS and the decrease in GSH content (Fig. 4I-K). Regression of the aggregate-to-monomer ratio and restoration of mitochondrial morphology after metformin treatment can be observed in the JC-1 staining results and transmission electron microscopy images (Fig. 4L-N). Correspondingly, the addition of CC again caused the increase in ROS levels and the decrease in GSH content, and the decrease in mitochondrial membrane potential was accompanied by abnormalities in the mitochondrial structure. Moreover, treatment with metformin alone did not affect the content of ROS and GSH, mitochondrial membrane potential, as well as mitochondrial morphology (Supplementary Fig. 3F-K). The aforementioned results suggest that metformin could effectively inhibit erastin-induced ferroptosis to improve the survival of NSCs, and inhibition of AMPK phosphorylation with compound C counteracted the ferroptosis inhibitory effect of metformin. Therefore, the protection of NSCs from ferroptosis by metformin is mediated by an AMPK-dependent mechanism.

### Metformin improves locomotor function of rats through AMPK activation after SCI

**Fig. 5:**
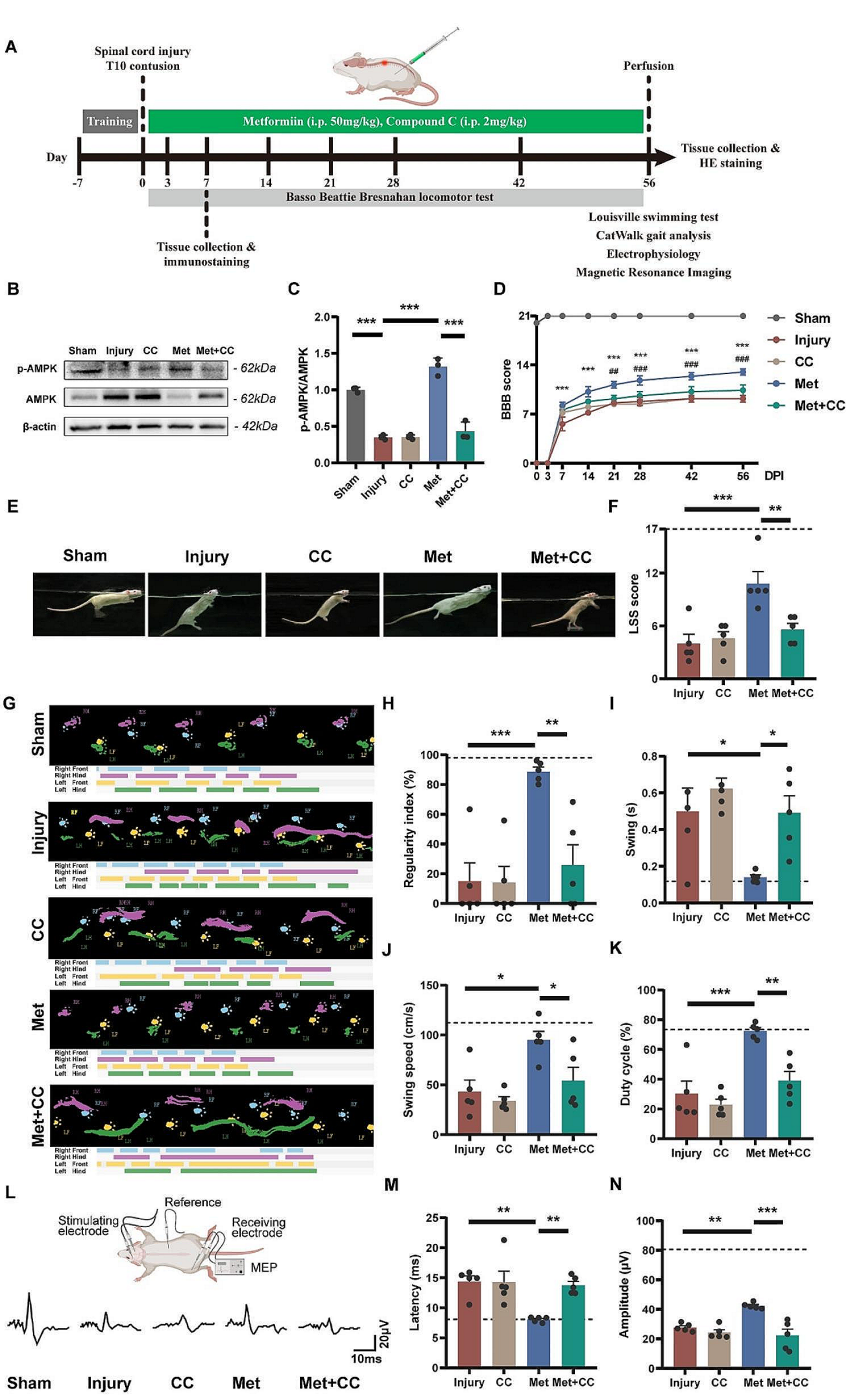
Metformin promotes functional recovery after SCI in rats via AMPK-dependent regulation. (**A**) Timeline of the experiments on spinal cord contusion rats. Spinal cord tissues were harvested at 7 days post-injury for immunofluorescence staining and western blot. Behavioral assessments were conducted until 56 days post-injury. (**B, C**) Representative western blot images of p-AMPK and AMPK and quantitative analysis of p-AMPK / AMPK expression level of spinal cords 7 days post-injury (*N* = 3). The relative expression level of target proteins was normalized by GAPDH and then calculated as fold changes of the sham group. (**D**) BBB scores at different time points after SCI (*N* = 5). (Injury vs. Met: ** *p* < 0.01, *** *p* < 0.001; Met vs. Met + CC: ^##^*p* < 0.01, ^###^*p* < 0.001) (**E, F**) Representative swimming images and quantitative analysis of LSS scores at 56 days post-injury (*N* = 5). The dotted line represents the baseline of the LSS score for the sham group. (**G**) Representative images of CatWalk footprint views and timing views at 56 days post-injury. (**H-K**) Quantitative analysis of regularity index, swing, swing speed and duty cycle shown in G (*N* = 5). The dotted line represents the baseline of the gait parameters for the sham group. (**L**) Schematic diagram of electrophysiology procedure and representative images of MEP at 56 days post-injury. (**M, N**) Quantitative analysis of the latency and amplitude of MEP shown in L (*N* = 5). The dotted line represents the baseline of the latency and amplitude of MEP for the sham group. The comparison of BBB scores was conducted using two-way ANOVA with Dunnett’s post-hoc test. Other analyses were conducted using one-way ANOVA with Dunnett’s post-hoc test. Data are shown as mean ± SEM. (* *p* < 0.05, ** *p* < 0.01, *** *p* < 0.001)

As mentioned above, metformin significantly promotes proliferation, neuronal differentiation and inhibits ferroptosis of NSCs via AMPK signaling pathway in vitro. To investigate the effects of metformin on endogenous NSCs following SCI and its potential for promoting functional recovery, we intraperitoneally injected metformin and AMPK inhibitor compound C into SCI rats, followed by behavioral assessments and histological examinations (Fig. 5A). The expression levels of AMPK signaling pathway of spinal cord tissues were assessed by western blot analysis. The phosphorylation level of AMPK in the Injury group was significantly lower compared to the Sham group (*p* < 0.001). And the phosphorylation level of AMPK in the Met group was significantly higher than that in the Injury group (*p* < 0.001). Furthermore, the application of Compound C completely abolished the effect of Metformin (*p* < 0.001), while the administration of Compound C alone did not affect the phosphorylation level of AMPK (Fig. 5B, C).

Behavioral assessments were conducted on rats at 0, 3, 7, 14, 21, 28, 42 and 56 days post-injury. From day 7 post-injury onwards, BBB scores of the Met group were significantly higher than those of the Injury group, and this trend persisted until 56 days post-injury (13.00 ± 0.45, 9.20 ± 0.49, respectively; *p* < 0.001) (Fig. 5D). Compared to the Met group, BBB scores of the Met + CC group were significantly lower at 56 days post-injury (13.00 ± 0.45, 10.40 ± 0.75, respectively; *p* < 0.001). The LSS swimming score, which mitigates the impact of animal weight, was used to assist in evaluating the recovery of hindlimb motor function in SCI rats [42]. The results showed that LSS score of the Met group was significantly higher than that of the Injury group and the Met + CC group (10.80 ± 1.36, 4.00 ± 1.05, 5.60 ± 0.68, respectively; *p* < 0.01) (Fig. 5E, F). Additionally, similar results were obtained from CatWalk gait analysis at 56 days post-injury. Rats in the Injury and Met + CC groups exhibited more hindlimb dragging, while the gait of rats in the Met group showed significant recovery. Parameters including regularity index, swing time, swing speed and duty cycle in the Met group (88.73 ± 3.02%, 0.14 ± 0.01 s, 95.09 ± 8.64 cm/s, 72.47 ± 2.27%) were significantly better than those in the Injury group (15.04 ± 12.31%, 0.50 ± 0.13 s, 43.17 ± 11.47 cm/s, 30.26 ± 8.57%) and the Met + CC group (25.85 ± 13.73%, 0.49 ± 0.09 s, 54.28 ± 13.33 cm/s, 39.07 ± 6.23%) (Fig. 5G-K). Compared to the Injury group, the CC group showed no significant differences in BBB scores, LSS scores, and CatWalk gait analysis results. Electrophysiological testing was employed to assess the recovery of motor nerve signal conduction in rats. Compared to the Injury group, the Met group exhibited a significantly shortened MEP latency (14.35 ± 0.98 ms, 8.05 ± 0.19 ms, respectively; *p* < 0.01) and increased wave amplitude (27.74 ± 1.19 µV, 42.37 ± 0.90 µV, respectively; *p* < 0.01), while these effects were reversed in the Met + CC group (Fig. 5L-N). The above results indicate that metformin facilitates the recovery of locomotor function and neural conduction following SCI, and these effects depend on the activation of AMPK.

### Metformin promotes proliferation and neuronal differentiation of endogenous NSCs through AMPK activation

**Fig. 6:**
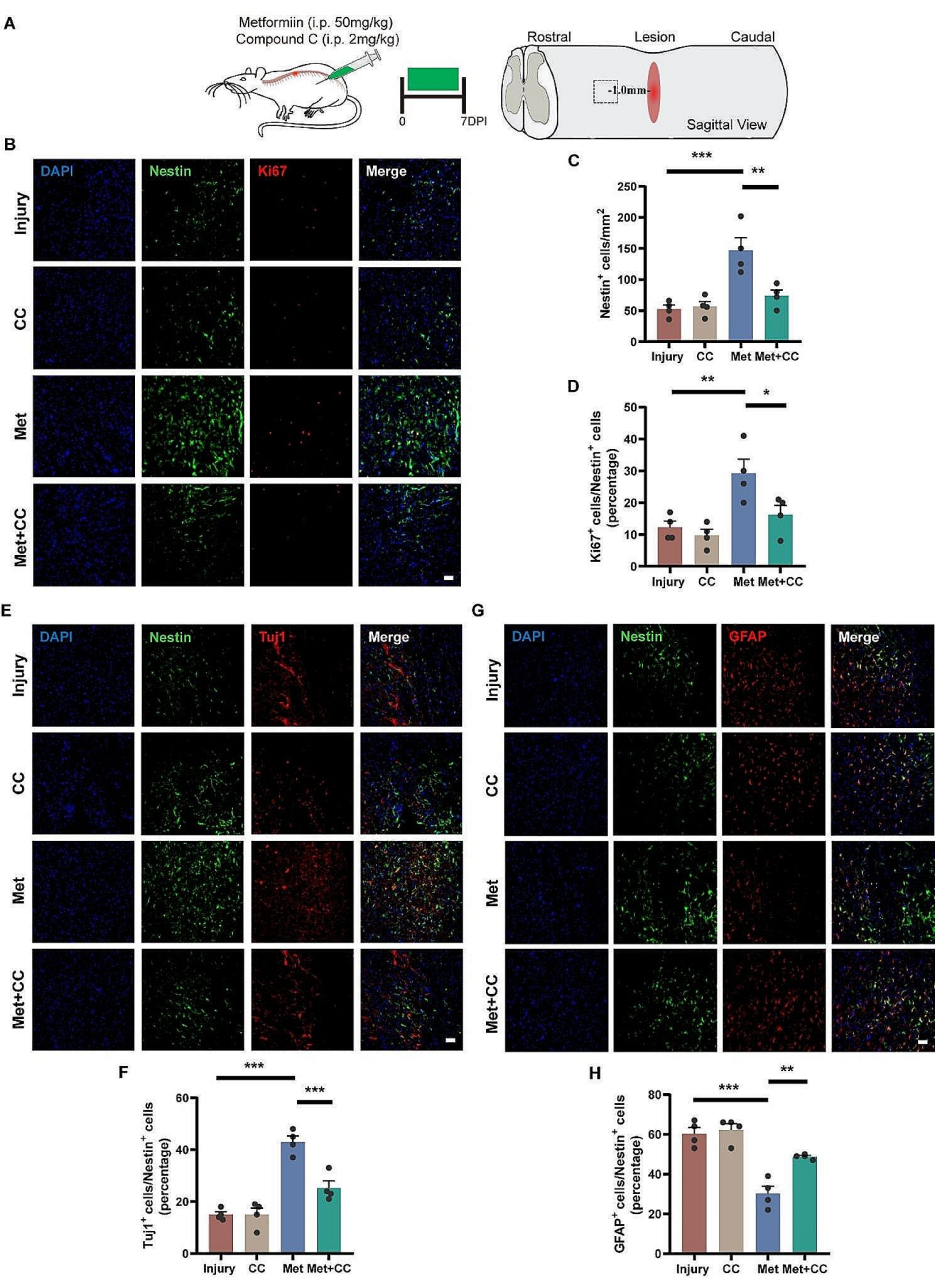
Metformin enhances endogenous NSCs proliferation and neural differentiation via AMPK-dependent regulation following SCI in rats. (**A**) Schematic diagram of the experiments on spinal cord contusion rats. Spinal cord tissues were harvested at 7 days post-injury and immunofluorescence staining was performed on coronal sections of spinal cord 1 mm rostral to the lesion center. (**B-D**) Representative immunofluorescence images of spinal cord sections double-labeled with Ki67 (red) and Nestin (green), and quantitative analysis of the number of Nestin positive cells and the proportions of Ki67 positive cells in Nestin positive cells (*N* = 4). Cell nuclei were stained with DAPI (blue). Scale bar = 100 μm. (**E, F**) Representative immunofluorescence images of spinal cord sections double-labeled with Tuj1 (red) and Nestin (green), and quantitative analysis of the proportions of Tuj1 positive cells in Nestin positive cells (*N* = 4). Cell nuclei were stained with DAPI (blue). Scale bar = 100 μm. (**G, H**) Representative immunofluorescence images of spinal cord sections double-labeled with GFAP (red) and Nestin (green), and quantitative analysis of the proportions of GFAP positive cells in Nestin positive cells (*N* = 4). Cell nuclei were stained with DAPI (blue). Scale bar = 100 μm. All analyses were conducted using one-way ANOVA with Dunnett’s post-hoc test. Data are shown as mean ± SEM. (* *p* < 0.05, ** *p* < 0.01, *** *p* < 0.001)

To validate whether metformin exerts similar effects on NSCs in the ependyma of spinal cord after injury, we conducted immunofluorescence staining 7 days post-injury to observe the endogenous neural regeneration at the injury site (Fig. 6A). Nestin and Ki67 were used as markers for NSCs and proliferating cells, respectively. The results showed that the number of Nestin-positive cells in the Met group was significantly higher than that in the Injury group and Met + CC group (147.30 ± 19.88 /mm^2^, 52.50 ± 6.40 /mm^2^, 74.00 ± 9.20 /mm^2^, respectively; *p* < 0.01) (Fig. 6B, C), and the proportion of Ki67-positive cells in Nestin-positive cells in the Met group was also significantly higher than that in other groups (29.25 ± 4.42%, 12.25 ± 1.97%, 16.25 ± 2.96%, respectively; *p* < 0.05) (Fig. 6B, D). Immunofluorescence staining was performed using Tuj-1 and GFAP as markers for neurons and astrocytes, respectively, co-stained with Nestin to observe the differentiation of NSCs in each group. The results showed that the proportion of Tuj-1-positive cells in Nestin-positive cells in the Met group was significantly higher than that in the Injury group and Met + CC group (43.00 ± 2.35%, 15.00 ± 1.08%, 25.25 ± 2.66%, respectively; *p* < 0.001) (Fig. 6E, F). Meanwhile, the proportion of GFAP-positive cells in Nestin-positive cells in the Met group was significantly lower than that in the other groups (30.25 ± 3.68%, 60.25 ± 3.20%, 48.75 ± 0.63, respectively; *p* < 0.01) (Fig. 6G, H). Additionally, there were no significant differences in the endogenous neural regeneration between the CC group and the Injury group. The above experimental results indicate that Metformin administration, dependent on AMPK signaling pathway, can enhance the proliferation of endogenous NSCs in the spinal cord and increase the proportion of neuronal differentiation, thereby increasing the number of regenerated neurons.

### Metformin inhibits endogenous NSCs ferroptosis in NSCs through AMPK activation

**Fig. 7:**
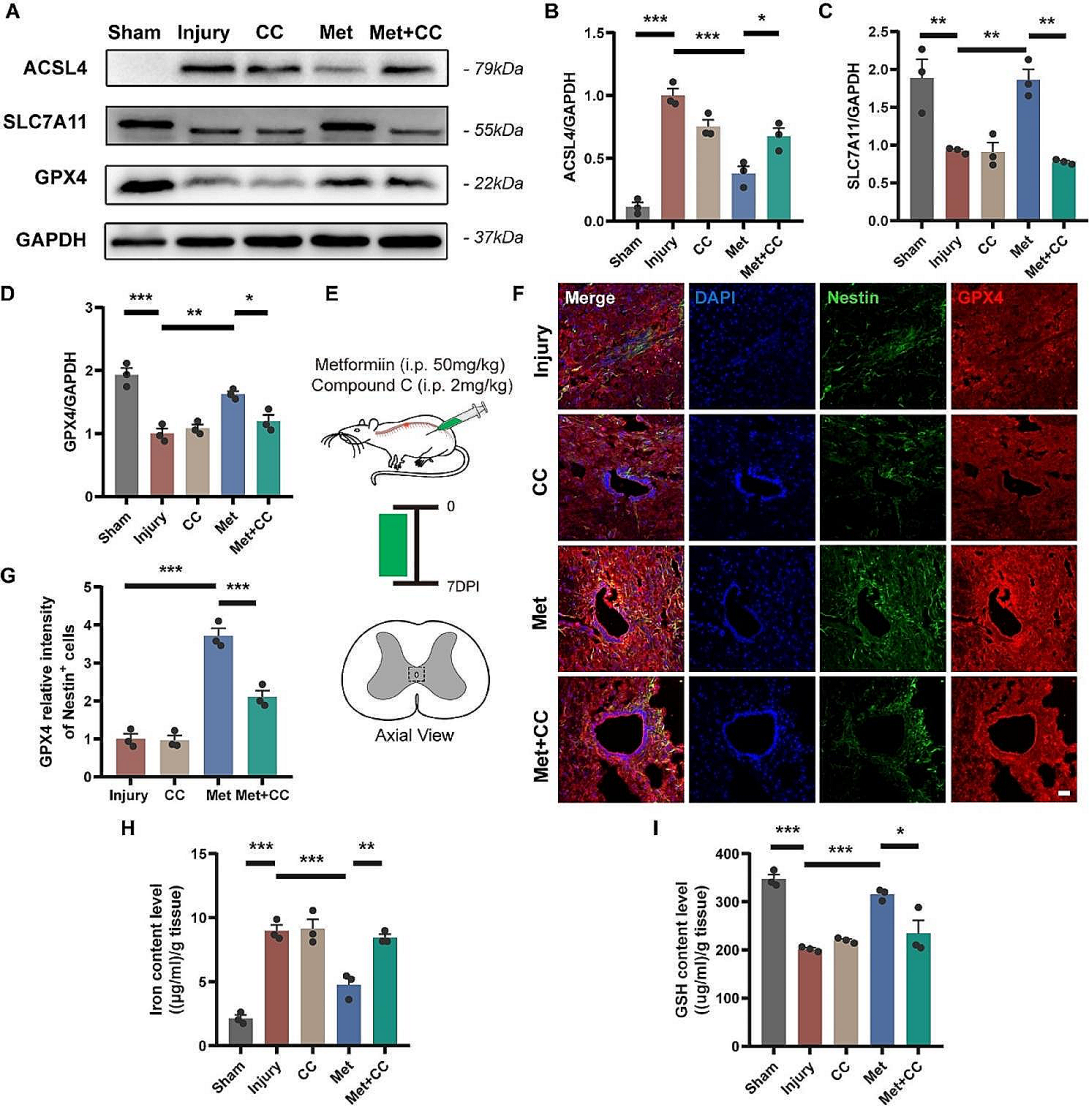
Metformin inhibits endogenous NSCs ferroptosis via AMPK-dependent regulation following SCI in rats. (**A-D**) Representative western blot images and quantitative analysis of expression level of ACSL4, SLC7A11 and GPX4 of spinal cord (*N* = 3). The relative expression level of target proteins was normalized by GAPDH and then calculated as fold changes of the sham group. (**E**) Schematic diagram of the experiments on spinal cord contusion rats. Spinal cord tissues were harvested at 7 days post-injury and immunofluorescence staining was performed on transverse sections of spinal cord 1 mm rostral to the lesion center. (**F, G**) Representative immunofluorescence images of spinal cord sections labeled with GPX4 (red), and quantitative analysis of the relative fluorescence intensity of GPX4 (*N* = 3). Cell nuclei were stained with DAPI (blue). The relative fluorescence intensity was calculated as fold changes of the injury group. Scale bar = 100 μm. (**H**) Quantitative analysis of iron content level of spinal cord (*N* = 3). (**I**) Quantitative analysis of GSH expression level of spinal cord (*N* = 3). All analyses were conducted using one-way ANOVA with Dunnett’s post-hoc test. Data are shown as mean ± SEM. (* *p* < 0.05, ** *p* < 0.01, *** *p* < 0.001)

To investigate the effects of metformin on ferroptosis of endogenous NSCs after SCI, we conducted relevant tests on rats 7 days post-injury. Western blot results showed a significant increase in ACSL4 protein expression and a significant decrease in SLC7A11 and GPX4 protein expression after injury, indicating the occurrence of ferroptosis in the injured spinal cord (Fig. 7A-D). The application of metformin reduced the expression of ACSL4 and increased the expression of SLC7A11 and GPX4, while the Met + CC group reversed the effects of metformin. GPX4 as a key marker of ferroptosis was used and co-stained with Nestin (Fig. 7E). The relative intensity of GPX4 in Nestin-positive cells in the Met group was significantly lower than that in the Injury group and the Met + CC group (Fig. 7F, G). Furthermore, consistent with the above trends, tissue iron content increased significantly after injury, while GSH content decreased (Fig. 7H, I). Metformin treatment resulted in decreased iron content (4.75 ± 0.58, 8.98 ± 0.45, respectively; *p* < 0.001) and increased GSH content (315.00 ± 6.78, 201.60 ± 2.76, respectively; *p* < 0.001), while the Met + CC group reversed the effects of metformin. There were no significant differences in the indicators of ferroptosis in rats treated with CC alone compared to the Injury group. These experimental results suggest that spinal cord injury can induce ferroptosis of endogenous NSCs, and the application of metformin can inhibit the occurrence of ferroptosis in the injured area, depending on the activation of AMPK.

### Metformin improves histological outcomes of spinal cord and bladder through AMPK activation

**Fig. 8:**
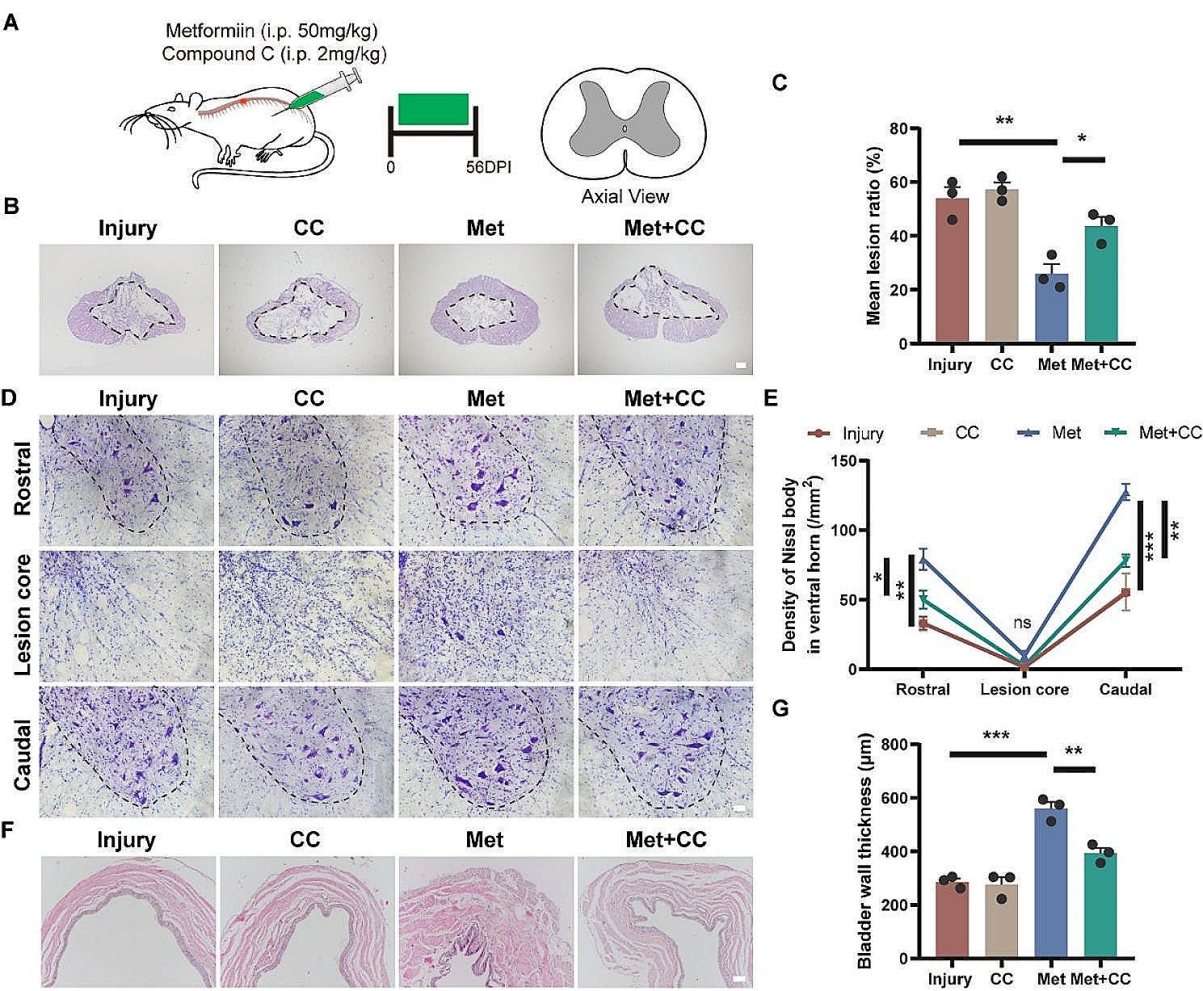
Metformin promotes tissue repair via AMPK-dependent regulation following SCI in rats. (**A**) Schematic diagram of the experiments on spinal cord contusion rats. Spinal cord tissues were harvested at 56 days post-injury and HE staining was performed on transverse sections of spinal cord lesion center. (**B, C**) Representative HE images of spinal cord sections, and quantitative analysis of the proportion of lesion area (*N* = 3). Scale bar = 200 μm. (**D, E**) Representative Nissl-stained images of spinal cord sections, and quantitative analysis of the number of Nissl bodies in ventral horn at rostral, lesion center and caudal site (*N* = 3). Scale bar = 50 μm. (**F, G**) Representative HE images of bladder sections, and quantitative analysis of the thickness of bladder wall (*N* = 3). Scale bar = 100 μm. All analyses were conducted using one-way ANOVA with Dunnett’s post-hoc test. Data are shown as mean ± SEM. (* *p* < 0.05, ** *p* < 0.01, *** *p* < 0.001, ns represents no statistical significance)

Next, we further validated the effectiveness of metformin in SCI repair from a histological perspective using HE staining and Nissl staining (Fig. 8A). HE staining revealed a significant reduction in cavity area in the Met group compared to the Injury group (26.00 ± 3.61%, 54.00 ± 4.16%, respectively; *p* < 0.01), while the cavity area in the Met + CC group exhibited a rebound (Fig. 8B, C). Nissl staining was performed on the ventral horn of the spinal cord in the rostral, lesion central, and caudal sections. The results indicated that the number of Nissl bodies in the Met group was significantly higher than that in the Injury group and the Met + CC group in the rostral and caudal sections, with no statistical differences observed among the groups in the lesion central section (Fig. 8D, E). Bladder wall thickness, which increases due to urinary retention-induced passive expansion, can serve as an effective auxiliary assessment indicator for bladder function recovery after SCI [43]. HE staining of the bladder revealed that the bladder wall thickness was significantly greater in the Met group compared to the Injury group (560.20 ± 24.44, 286.40 ± 12.59, respectively; *p* < 0.001), while it was significantly lower in the Met + CC group compared to the Met group (393.10 ± 19.91, 560.20 ± 24.44, respectively; *p* < 0.01) (Fig. 8F, G). These findings suggest that Metformin-dependent AMPK activation promotes histological improvements in the spinal cord and bladder following SCI.

## Discussion

The substantial loss of neurons stands as a primary impediment to achieving effective functional recovery following SCI, and the activation level of endogenous NSCs and their differentiation into neurons directly affect the recovery process [44, 45]. The findings of this study underscore that metformin significantly enhances both the proliferation and neuronal differentiation of NSCs. Establishing an NSCs ferroptosis model through erastin treatment, metformin effectively mitigates the extent of NSCs ferroptosis. Leveraging these neuroregenerative and neuroprotective properties, metformin holds promise in fostering the recovery of hind limb locomotor function in SCI rats. It is worth noting that the beneficial effects mentioned above are dependent on the activation of AMPK.

Metformin, as a highly safe biguanide medication, is currently regarded as the first-line oral drug for type 2 diabetes [46]. Apart from its glucose-lowering effects, metformin has shown significant progress in the treatment of cancer, cardiovascular diseases, and kidney disorders [47–50]. In recent years, the potential application of metformin in neurological disorders, including stroke, Parkinson’s disease, multiple sclerosis, epilepsy, and central nervous system trauma, has attracted considerable attention [48–53]. The multifunctional nanospheres carrying metformin, developed by Yuan et al., have been demonstrated to inhibit inflammatory responses and the elevation of oxidative stress levels, thereby promoting the recovery of locomotor function after SCI [54]. However, the effects of metformin on endogenous NSCs remain unclear. Therefore, our study explored the impact of metformin on the proliferation, differentiation, and ferroptosis of endogenous NSCs during SCI repair through in vitro and in vivo experiments, further highlighting the role and molecular mechanisms of metformin in neuroregeneration and neuroprotection.

Developing effective treatment strategies for central nervous system trauma remains a significant challenge, with stem cell transplantation and modulation of endogenous neuroregeneration being the most promising repair approaches. However, the activation capacity of NSCs is limited following SCI, and both transplanted and endogenous NSCs tend to differentiate into glial cells rather than neurons. Previous studies have found that metformin promotes neurogenesis in rodent cortical precursors via the atypical PKC-CBP pathway [55]. Michael Fatt et al. reported that metformin promotes adult NSCs proliferation which is dependent on the p53 family member [33]. In addition, metformin has been observed to expand the size of the neural precursor cells pool and promote differentiation in childhood brain injury and adult female stroke models, although its mechanism of action remains unclear [32, 56, 57]. Here, we found that treatment with 1 µM metformin significantly enhanced NSCs proliferation, neural differentiation, and reduced differentiation into astrocytes, but these effects were counteracted when compound C was used together. Therefore, these results suggest that metformin activates NSCs to increase the number of neurons via the canonical AMPK-dependent mechanism, contrary to previous reports that suggested that these effects are regulated by noncanonical targets of action.

The inhibitory microenvironment of the lesion core caused by secondary injury hinders the survival of endogenous and transplanted NSCs, posing a significant challenge for SCI repair. Recent studies have highlighted the role of ferroptosis, characterized by iron accumulation and lipid peroxidation, in the pathophysiological processes following SCI [24]. Following the primary injury, iron accumulation occurs at the lesion site and it is mainly due to the release of iron from hemoglobin in the red blood cells [20, 21]. Subsequently, iron accumulation leads to the generation of excess free radicals and mediates lipid peroxidation, ultimately leading to ferroptosis [58–60]. Ferroptosis inhibitors have demonstrated neuroprotective effects in various central nervous system trauma models [61–64]. A previous study reported that metformin could kill cancer cells by inducing ferroptosis in oncology treatment [65]. Controversially, recent evidence suggests that AMPK activation promotes tumor progression by inhibiting ferroptosis in certain tumor types [66–68]. Besides, metformin has been found to alleviate subchondral sclerosis in a mouse osteoarthritis model by inhibiting ferroptosis [69]. However, the promotive or inhibitory effects of metformin on ferroptosis in NSCs have not been previously reported. In this study, we successfully developed a ferroptosis model of NSCs with 100 µM erastin. Moreover, metformin was observed to reverse the changes in the expression of key ferroptosis-related proteins ACSL4, SLC7A11, and GPX4 in NSCs. In addition, metformin increased GSH synthesis to reduce ROS production and improved mitochondrial membrane potential and mitochondrial morphology, thereby increasing the survival of NSCs. The above-mentioned effects were abolished by using compound C to inhibit metformin-mediated AMPK activation. These results suggest that metformin could inhibit ferroptosis in NSCs and this is also regulated by classical AMPK-dependent mechanisms.

This study also has certain limitations. Previous research has shown that metformin has multi-target effects, inhibiting inflammation and promoting M2 polarization in microglia/macrophages [70]. However, in this study, metformin was administered via intraperitoneal injection, lacking specific targeting, and thus unable to determine the roles of microglia/macrophages or other cells in the spinal cord repair process. Secondly, oligodendrocytes play a crucial role in remyelination and neuronal survival [71]. However, this study focused only on the differentiation of neurons and astrocytes from neural stem cells. Future experiments are needed to explore the differentiation direction and function of oligodendrocytes. Furthermore, due to the potential off-target effects of erastin, further investigation is needed to use additional ferroptosis inducers to establish a more robust and comprehensive NSC ferroptosis model. Additionally, this study found that the effects of metformin on neural stem cells are dependent on the AMPK signaling pathway. However, only inhibitors were used in this study for validation. Future research should employ gene silencing and transgenic animal models for in vitro and in vivo validation.

## Conclusions

**Fig. 9:**
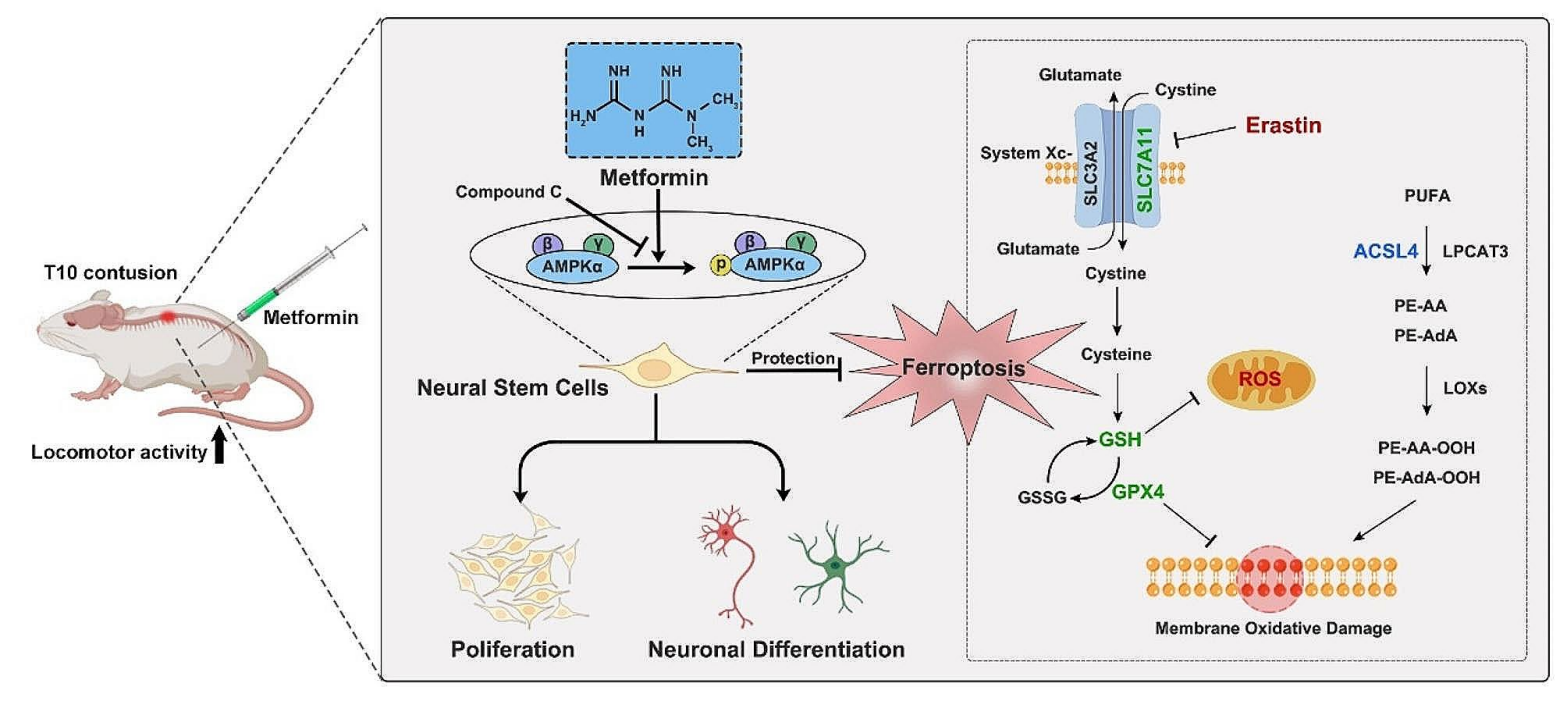
Schematic illustration of the effects of metformin on NSCs fate and functional recovery following SCI in rats. Metformin enhances the proliferation and neuronal differentiation of NSCs and effectively inhibits ferroptosis to promote the recovery of locomotor function in rats after SCI. Moreover, these beneficial effects of metformin on NSCs are regulated by AMPK-dependent mechanism

In summary, our findings demonstrate that metformin significantly enhances the proliferation and neuronal differentiation of NSCs both in vitro and in vivo. Furthermore, we established an in vitro ferroptosis model of NSCs using erastin treatment, and metformin effectively improves cell survival by inhibiting ferroptosis. Notably, these beneficial effects are contingent upon the activation of AMPK. Overall, metformin, dependent on AMPK activation, promotes the recovery of locomotor function after SCI by modulating the proliferation, differentiation fate, and inhibiting ferroptosis in endogenous NSCs (Fig. 9). These findings suggest that metformin holds promise as a candidate for SCI repair by promoting nerve regeneration and neuroprotection.

### Electronic supplementary material

Below is the link to the electronic supplementary material.

Supplementary Material 1

## Data Availability

All data are available from the corresponding author on reasonable request.

## Abbreviations

SCI: Spinal cord injury
NSCs: Neural stem cells
AMPK: Adenosine 5’-monophosphate-activated protein kinase
Glutathione peroxidase 4: GPX4
SLC7A11: Solute carrier family 7 member 11
ACSL4: Acyl-coenzyme A synthetase long-chain family member 4
GSH: Glutathione
ROS: Reactive oxygen species
MMP: Mitochondrial membrane potential
HE: Hematoxylin-eosin
BBB: Basso, Beattie, and Bresnahan
LSS: Louisville swim scale
MEP: Motor evoked potentials
