# Supplementary material for: Metformin enhances endogenous neural stem cells proliferation, neuronal differentiation, and inhibits ferroptosis through activating AMPK pathway after spinal cord injury
Source: J Transl Med. 2024 Aug 5;22:723. doi: 10.1186/s12967-024-05436-9 (PMC11302024; doi:10.1186/s12967-024-05436-9)
Supplement: Supplementary file 1 — Supplementary Material 1 [file 12967_2024_5436_MOESM1_ESM.docx]

**Supplementary Table 1 The group of each experiment and its sample size.**

| **Methods** | **Sample size** | | | | |
| --- | --- | --- | --- | --- | --- |
|  | **Sham** | **Injury** | **CC** | **Met** | **Met+CC** |
| **BBB score** | 5  5  5  5 | 5 | 5 | 5 | 5 |
| **LSS score** |  | 5 | 5 | 5 | 5 |
| **CatWalk gait analysis** |  | 5 | 5 | 5 | 5 |
| **Electrophysiological testing** |  | 5 | 5 | 5 | 5 |
| **HE and Nissl staining** | - | 3 | 3 | 3 | 3 |
| **IF staining (Nestin/Ki67/Tuj1/GFAP)** | - | 4 | 4 | 4 | 4 |
| **IF staining (Nestin/GPX4)** | - | 3 | 3 | 3 | 3 |
| **Western blot** | 3 | 3 | 3 | 3 | 3 |
| **Tissue iron detection** | 3 | 3 | 3 | 3 | 3 |
| **GSH detection** | 3 | 3 | 3 | 3 | 3 |

The rats used for BBB scoring, LSS scoring, CatWalk gait analysis, electrophysiological tests, HE staining, and Nissl staining were all from the same batch.

**Supplementary Table 2 The information of antibodies.**

| **Antibody** | **Source** | **Catalog No.** | **Applications** |
| --- | --- | --- | --- |
| Nestin | R&D systems | MAB2736 | Immunofluorescence |
| SOX2 | Abcam | ab93689 | Immunofluorescence |
| Tuj-1 | Abcam | ab18207 | Immunofluorescence |
| GFAP | Cell Signaling Technology | 3670 | Immunofluorescence |
| Olig2 | R&D systems | AF2418 | Immunofluorescence |
| p-AMPK | Cell Signaling Technology | 2535 | Western blot |
| AMPK | Cell Signaling Technology | 2793 | Western blot |
| ACSL4 | Abcam | ab155282 | Western blot |
| SLC7A11 | Abcam | ab175186 | Western blot |
| GPX4 | Abcam | ab125066 | Western blot |
|  |  |  | Immunofluorescence |
| GAPDH | Proteintech | 10494-1-AP | Western blot |
| Goat Anti-Rabbit Alexa Fluor 555 | Abcam | ab150086 | Immunofluorescence |
| Goat Anti-Mouse Alexa Fluor 488 | Abcam | ab150117 | Immunofluorescence |
| Donkey Anti-Rabbit Alexa Fluor 647 | Abcam | ab150075 | Immunofluorescence |
| Donkey Anti-Mouse Alexa Fluor 488 | Abcam | ab150105 | Immunofluorescence |
| Donkey Anti-Goat Alexa Fluor 555 | Abcam | ab150130 | Immunofluorescence |
| HRP-labeled Goat Anti-Rabbit | Beyotime | A0208 | Western blot |
| HRP-labeled Goat Anti-Mouse | Beyotime | A0216 | Western blot |


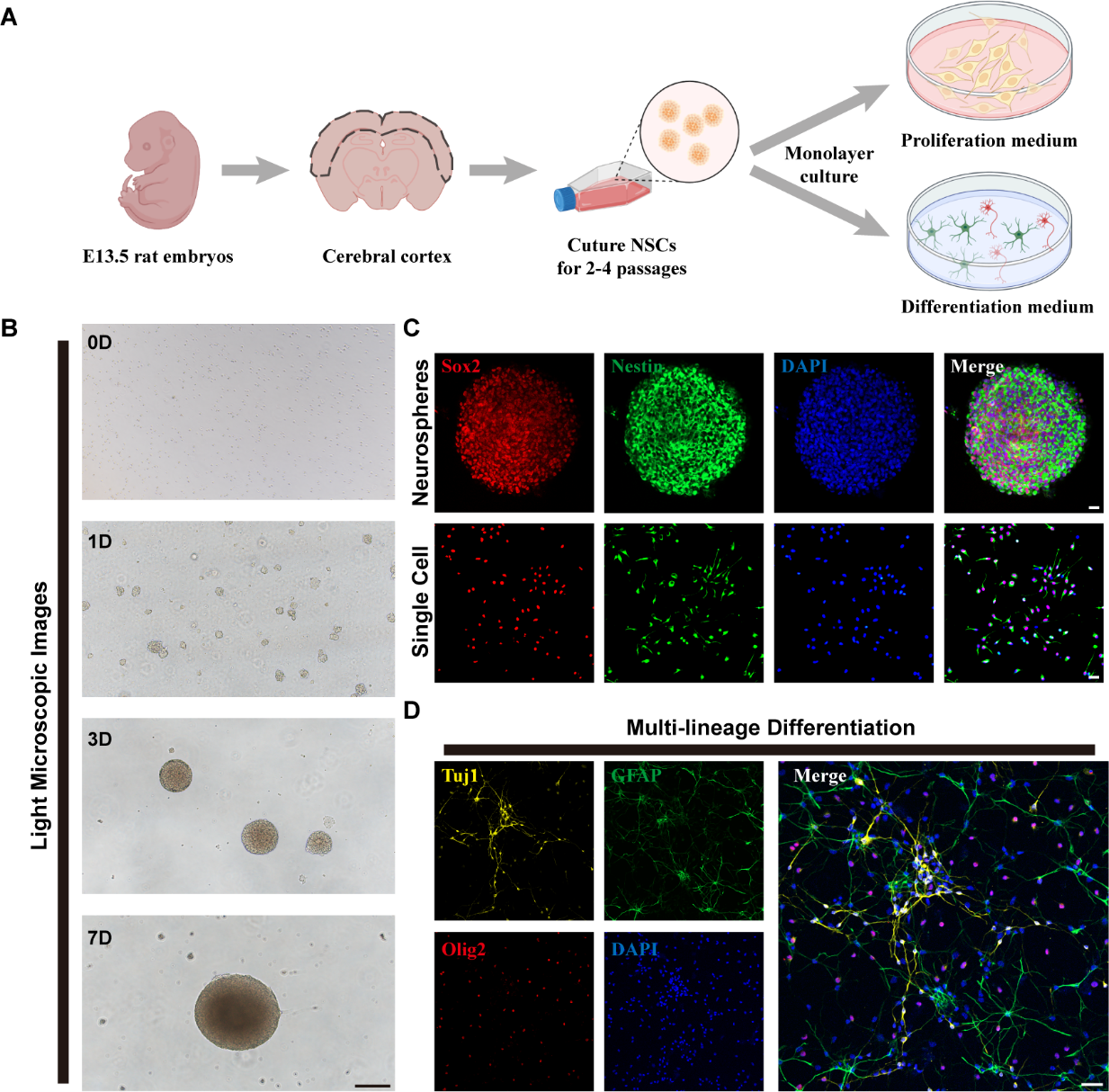


**Supplementary Figure 1** **Rat embryonic cortical NSCs proliferate and tri-lineage differentiates. A** Schematic diagram of embryonic cortical NSCs extraction and culture. **B** Representative phase contrast microscope images of primary NSCs cultured in growth-medium on days 0, 1, 3, and 7. Scale bar = 200 μm. **C** Representative immunofluorescence images of NSCs double-labeled with SOX2 (red) and Nestin (green) on day 7. Cell nuclei were stained with DAPI (blue). Scale bar = 20 μm. **D** Representative immunofluorescence images of NSCs triple-labeled with Tuj-1 (yellow), GFAP (green), and Olig2 (red) on day 7 after changing to differentiation medium. Cell nuclei were stained with DAPI (blue). Scale bar = 20 μm.


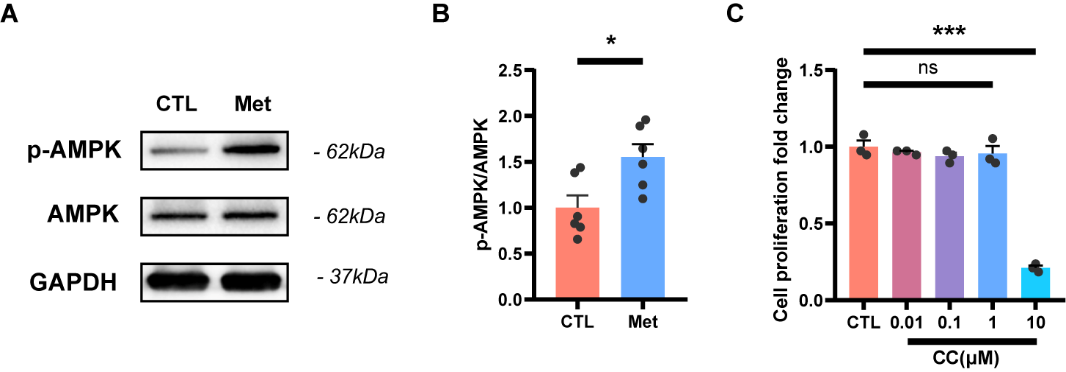


**Supplementary Figure 2 Metformin activates AMPK signaling pathway in NSCs. A, B** Representative western blot images of p-AMPK and AMPK and quantitative analysis of p-AMPK / AMPK expression level in metformin-treated and untreated NSCs (N=6). The relative expression level of target proteins was normalized by GAPDH and then calculated as fold changes of the control group. **C** Cell viability assay of NSCs treated with different concentrations of compound C (N=3). The cell viability was calculated as fold changes of the control group. B was analyzed using unpaired t-tests, and C was analyzed using one-way ANOVA and Tukey's post-hoc test. Data are shown as mean ± SEM. (* *p* < 0.05, ** *p* < 0.01, *** *p* < 0.001, ns represents no statistical significance).


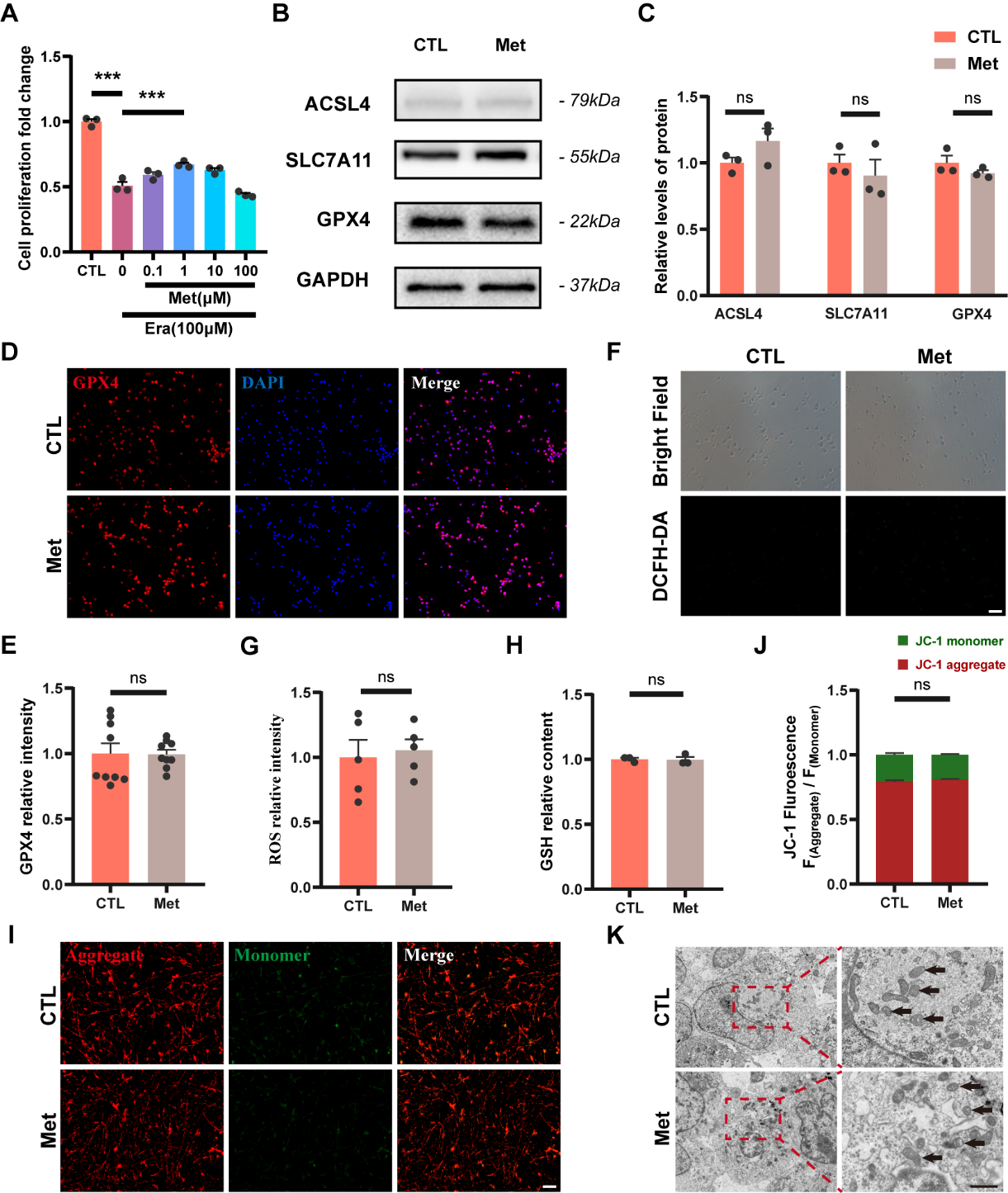


**Supplementary Figure 3 Metformin has no effect on ferroptosis of NSCs without erastin. A** Cell viability assay of NSCs with different treatments (N=3). The cell viability was calculated as fold changes of the control group. **B, C** Representative western blot images and quantitative analysis of expression level of ACSL4, SLC7A11 and GPX4 in NSCs (N=3). The relative expression level of target proteins was normalized by GAPDH and then calculated as fold changes of the control group. **D, E** Representative immunofluorescence images of NSCs labeled with GPX4 (red), and quantitative analysis of the relative fluorescence intensity of GPX4 (N=9). Cell nuclei were stained with DAPI (blue). The relative fluorescence intensity was calculated as fold changes of the control group. Scale bar = 50 μm. **F, G** Representative images of DCFH-DA and quantitative analysis of the relative fluorescence intensity of ROS in NSCs (N=5). The relative fluorescence intensity was calculated as fold changes of the control group. Scale bar = 50 μm. **H** Quantitative analysis of GSH expression level of NSCs (N=3). The relative GSH content was calculated as fold changes of the control group. **I, J** Representative immunofluorescence images of JC-1 and quantitative analysis of the relative fluorescence intensity ratios of aggregate (red) / monomer (green) (N=6). Scale bar = 50 μm. **K** Representative transmission electron microscopy images of NSCs. Mitochondria were indicated by black arrows. Scale bar = 1 μm. A was analyzed using one-way ANOVA with Dunnett’s post-hoc test, and all other analyses were conducted using unpaired t-tests. Data are shown as mean ± SEM. (* *p* < 0.05, ** *p* < 0.01, *** *p* < 0.001, ns represents no statistical significance).
